# Monitoring Universal Health Coverage (UHC) in high Tuberculosis burden countries: Tuberculosis mortality an important tracer of UHC service coverage

**DOI:** 10.1371/journal.pone.0223559

**Published:** 2019-10-30

**Authors:** Michael Reid, Glenna Roberts, Eric Goosby, Paul Wesson

**Affiliations:** 1 University of California, San Francisco, School of Medicine, San Francisco, California, United States of America; 2 University of California, San Francisco, Institute for Global Health Sciences, San Francisco, California, United States of America; 3 University of California, San Francisco, Center of AIDS Prevention Studies, San Francisco, California, United States of America; UCIBIO-REQUIMTE, Faculty of Pharmacy, University of Porto, PORTUGAL

## Abstract

**Background:**

There is a paucity of empiric data evaluating whether Tuberculosis (TB) is a useful surrogate measure for Universal Health Coverage (UHC), despite recognition of the importance of TB control efforts as part of the broader UHC agenda. We hypothesized that indicators of TB burden and coverage are sensitive tracers of UHC, when compared to other disease-specific indicators of service provision.

**Methods:**

Linear regression models were used to determine the extent to which variability in UHC Service Coverage Index (SCI) was accounted for by (1) TB incidence rates and (2) TB mortality rates across 183 countries. Dominance analyses, stratifying countries by World Bank income criteria and TB burden, were used to determine the importance of TB treatment coverage in predicting UHC SCI scores, relative to other disease-specific indicators of service provision.

**Results:**

Across 183 countries, TB incidence rate and TB mortality rate were negatively correlated, with UHC SCI score, (r = -0.67 and r = -0.74, respectively). In linear regression models including all 183 countries, TB incidence rates explained 45% of the variability in SCI scores; TB mortality rate explained 55% of variability. Restricting models to the 30 highest TB burden countries, both incidence and mortality explained less of the variability in SCI score (16% and 36%, respectively). In dominance analysis, comparing 13 disease-specific indicators of service provision, TB effective treatment coverage, ranked ninth overall. In dominance analysis stratified by TB burden, the TB treatment coverage estimate was ranked ninth in the 30 high burden countries and sixth in the 153 non-high burden countries. In separate analyses stratified by world bank income status, TB coverage ranked as third most important variable in LICs and fifth in LMICs and UMICs, but was less important in analysis restricted HICs (ranked seventh).

**Conclusions:**

Compared to other disease-specific indicators of service provision, TB coverage was an important indicator of overall UHC service coverage, especially in low-income countries. These findings highlight that national-level inequities in TB-coverage may be an important tracer of universal health coverage.

## Introduction

Universal health coverage (UHC) is the goal that all people receive the essential health services that they need, without being exposed to financial hardship.[1,2] It is a central target of the Sustainable Development Goals (SDG).[3] While there are numerous proposed strategies for achieving UHC[4] the objectives of UHC are the same, regardless of approach: improving access to health services, improving the health of individuals covered and providing financial risk protection.[5] Recent reports have sought to determine which health interventions should be included in any definition of UHC, especially because many low and middle income countries have limited resources to deliver the breadth of services offered in many high income settings.[6,7] Furthermore, much work has been done to determine what the cost of implementing an essential UHC package will look like for countries of different income categories.[1,2,6] Unfortunately, data describing country progress towards implementing an essential UHC package of services is sparse, limited by data quality and diversity of definitions of what range of indicators of service coverage would be most representative.

Recently, World Health Organization (WHO) and the World Bank proposed the use of a UHC Service Coverage Index (UHC SCI) tool to assess country-coverage with a range of different interventions. [5] This tool summarizes country-level coverage of 16 essential service indices across four core domains; (a) reproductive, maternal, newborn and child health, (b) infectious diseases, (c) non-communicable diseases and (d) service capacity and access, among the general population and the most disadvantaged populations.[5] While, the SCI offers a useful means of comparing essential health coverage across countries, the index is challenging to calculate, based on asynchronously collected and highly variable data, and offers limited utility for tracking prospective progress on quality or coverage.

By contrast, indicators of tuberculosis (TB) burden, in particular TB incidence and mortality, are reported annually for all countries providing accurate information on trends in disease burden.[8] Tuberculosis disproportionately impacts vulnerable communities, and trends in TB incidence and mortality are also sensitive surrogates of health inequity.[2,9] Thus, geographical and financial access barriers for general health services are invariably access barriers for TB services as well. We hypothesize that indicators of TB epidemiologic control can serve as useful, simple surrogates for more complex indices of UHC, such as the UHC SCI, especially in countries with a high TB burden. To determine if this is the case, we evaluated the extent to which TB incidence and mortality explain the variability in UHC SCI scores across low and high burden countries.

## Methods

### Calculation of UHC SCI score

The rationale and methodology for how UHC SCI is derived has been described in depth elsewhere.[5] Briefly, the index is constructed from geometric means of 16 tracer indicators within each of four categories: (a) reproductive, maternal and new born and child health, (b) infectious disease control, (c) non-communicable diseases and (d) service capacity and access. Each indicator is calculated based on publicly reported data and a determination of the best way of assessing disease-specific service coverage at country-level, with a standardized approach applied across all countries. For each country, all 16 indices were aggregated and reported as a score, measured on a scale of 0–100%, with 100% representing achievement of universal health coverage. The TB-specific SCI sub-index, referred to as the *TB effective coverage score*, combines the rate of case detection and of treatment success to estimate the proportion of all people with TB who successfully complete treatment. Calculation of the case detection rate requires estimates of incident cases (including those not identified by the health-care system), which is reported annually in the WHO Global TB report. Treatment success is measured through administrative data, and includes all patients who successfully completed treatment without bacteriological evidence of treatment failure. Both are reported by all countries annually and reported in the Global TB report, published by WHO.[8]

Notably, indicators within the service capacity and access category, including the hospital bed density and health worker density, have a lower bound of 0 but do not have a clear optimal level of maximum. For these variables, a threshold value was selected based on observed minimum values across high-income Organization for Economic Co-operation and Development (OECD) countries. Countries with values above the thresholds for these indicators where held at 100 and those below were linearly rescaled between 0 and 100.

### Analyses to describe relation between TB indices and UHC SCI

UHC SCI scores were calculated for all United Nations (UN) member states based on methodology described by Hogan et al and using WHO 2015 data.[5] Time-concurrent TB incidence and mortality data for all 183 UN member states were sourced from the Global TB report.[8] Descriptive analyses including T-test (for continuous variables) and Kruskal-Wallis test (for comparison of medians) were used to compare UHC SCI scores across World Bank Income groups[10] (High-Income Countries [HICs], Upper Middle-Income Countries [UMICs], Middle-Income Countries [MICs], Lower Middle-Income countries [LMICs] and Low-Income Countries [LICs]), and to compare countries based on TB burden.[8] The 30 ‘highest burden countries’ were defined using the current WHO definition, and included the 20 high TB burden countries based on absolute number of incident cases and the ten high TB burden countries based on severity of disease burden in terms of incidence per capita.[8] Pearson correlation coefficients were calculated to assess the associations between TB indicators and the UHC SCI across all countries, stratified by TB burden. ANOVA model test was used to assess the association of the service coverage index across World Bank income groupings, and between high and non-high burden TB countries.

Linear regression models were used to examine the association between UHC SCI scores and (1) national TB incidence rates and (2) national TB mortality rates, in all countries. In additional models restricted to high and non-high TB burden countries, regression analyses were also performed to examine UHC SCI score and (1) TB incidence and (2) TB mortality. Because both TB incidence and TB mortality rates were found to have a left skewed distribution, they were log-transformed to normalize their distributions. The R-squared values for models were compared to determine the extent to which variability in UHC SCI score was explained by each of the TB indicators. As a sensitivity analysis, each of these regression models were repeated using a modified UHC SCI score, from which the TB sub-index score had been dropped, in order to assess for and mitigate potential collinearity.

### Dominance analyses

To determine the relative importance of TB to the overall UHC SCI score we then performed a ‘dominance analysis.’ This is a pairwise regression approach that tests all the indicators against one another as a measure of predictor ‘importance’ in terms of their contribution to the UHC SCI score, in order to evaluate each indicator’s ability to predict the UHC SCI score. [11] Importance was determined by the size of the coefficient without any inference on the relative ‘significance’ of each indicator variable. Dominance analysis was performed for all countries and then stratified by TB burden status and by World Bank income categories. We determined that traditional multivariate regression was not appropriate for assessing ‘importance’ in this analysis due to multiple collinearity (i.e. when all predictors are correlated).

Indicators for malaria and cancer screening coverage, as well as essential medications, were not included in this analysis due to lack of consistent reporting for the majority of United Nations member states. All analyses were conducted using Stata version 14 (College Station, TX).

## Results

**Fig 1:**
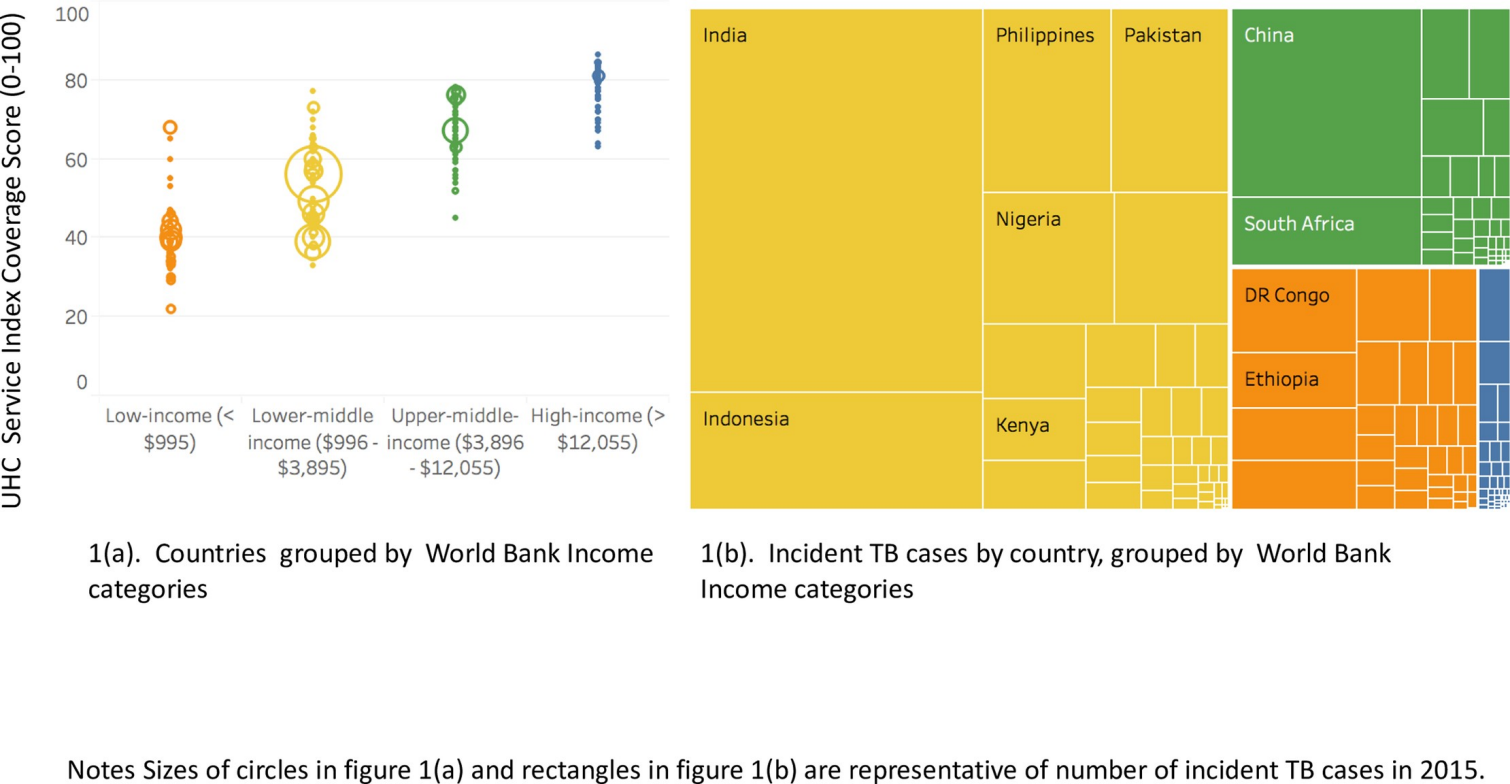
**(a).** UHC Service coverage index scores, stratified by World Bank country income categories and **1(b)** TB burden delineated by absolute number of incident cases (2015) and World Bank country income categories.

Across 183 countries, the median service coverage index was 65 (Interquartile range [IQR]: 48–75), with 44 countries having UHC SCI scores greater than 75 of which 35 were classified as HICs, according to World Bank criteria. There were 45 countries in the lowest quartile, of which 40 were classified as LICs. Comparing median UHC SCI scores, there was a significant difference in UHC SCI across World Bank country income groups, with median service coverage scores lowest in LICs (39.5, IQR 34–46) and highest in the HICs (79 [IQR:73, 81], p<0.001). UHC SCI scores were also significantly lower in the thirty highest TB burden countries compared to non-high TB burden countries (46.5 [IQR: 39–58] vs. 69 [IQR: 61 vs. 77], p<0.001). As illustrated in Fig 1, the burden of TB, defined by incident cases in 2015, was highest in LMICs.

### Univariable analyses to assess the association of TB indicators with UHC SCI

Pearson correlation coefficients evaluating the association of TB incidence and TB mortality rates, and the UHC SCI were high and negatively correlated, in analysis that included all countries (r = -0.67 and r = -0.74, respectively) (Table 1). In analyses stratified by TB burden (using the WHO definition), the correlations were higher in non-high TB burden countries, compared to high TB burden countries.

**Table 1:** Pearson correlations for fifteen service coverage indicators, TB incidence and TB mortality, with UHC Service Coverage Index Score across 183 countries.

	All countries (n = 183)	High TB burden countries (n = 30)	Non-High TB burden countries (n = 153)
**Tuberculosis effective treatment**^**a**^	0.56	0.62	0.53
**TB Incidence (2015 rates)**	-0.67	-0.22	-0.56
**TB mortality (2015 rates)**	-0.74	-0.36	-0.59
Family planning^b^	0.73	0.83	0.77
Pregnancy and delivery care^c^	0.79	0.60	0.80
Child immunization^d^	0.59	0.61	0.58
Child treatment^e^	0.75	0.66	0.74
HIV treatment^f^	0.49	0.46	0.48
Water and sanitation^g^	0.88	0.83	0.87
Prevention of cardiovascular disease^h^	0.69	0.59	0.70
Management of diabetes^i^	-0.13	0.05	-0.08
Tobacco Control^j^	-0.25	-0.25	-0.25
Hospital access^k^	0.72	0.66	0.71
Health care worker density^l^	0.87	0.80	0.87
Health security^m^	0.70	0.73	0.72

^a^Tuberculosis treatment–measured as TB effective treatment, calculated as ration of rate of case detection to rate of TB treatment

^b^ Family planning–measured as demand satisfied with modern methods in women aged 15–49 years who are married or in a union (%)

^c^ Pregnancy and delivery care–measured as four or more visits to antenatal care (%)

^d^ Child immunization–measured as children aged 1 year who have received three doses of diphtheria, tetanus and pertussis vaccine (%)

^e^ Child treatment–measured as care-seeking behavior for children with suspected pneumonia (%)

^f^ HIV treatment–measured as the proportion of people with HIV receiving antiretroviral treatment (5)

^g^ Water and sanitation—measured as the proportion of households with access to at least basic sanitation (%)

^h^ Prevention of cardiovascular disease–measured as prevalence of non-raised blood pressure regardless of treatment status (%)

^i^ Management of diabetes–measured as mean fasting plasma glucose measured in country-specific household surveys

^j^ Tobacco control–measured as adults aged 15 years or older who had not smoked tobacco in the previous 30 days (%)

^k^ Hospital access—measured as the number of hospital beds per person in each country

^l^Health care worker density—measured as the number of health professionals per person, comprising physicians, psychiatrists and surgeons

^m^ Health Security–measured based on International Health regulations core capacity index. **Notes**: Malaria prevention is also included in the UHC SCI for countries where malaria is prevalent. Since most countries do not collect this data, we excluded it from our analysis. Cervical cancer screening and access to essential medicines were excluded because of low data availability.

### Linear regression analyses to assess the association of TB indicators with UHC SCI

We next evaluated the association of each independent variable with UHC SCI in separate regression models stratified according to whether countries are TB high burden (n = 30) or non-high burden (n = 133). Examining the association between UHC SCI and log-transformed TB incidence rate, the R-squared value was 0.45 in models including all 183 countries, 0.16 in models restricted to high TB burden countries only, and 0.44 in non-high burden countries. Examining the association between UHC SCI and log-transformed TB mortality rate, the R-squared value was 0.55 in models including all 183 countries, 0.36 in models restricted to high burden countries only, and 0.54 in non-high burden countries (Table 2). All of these models were statistically significant with p-values <0.05.

**Table 2:** Multivariate regression analysis assessing association of TB incidence rate and TB mortality rate with UHC SCI scores; multivariate models stratified based on TB burden.

	UHC service coverage Index		
	All countries (n = 183) R square Value	High TB burden countries (n = 30) R square Value	Non-High TB burden countries (n = 153) R square Value
TB Incidence rate (2015)	0.45	0.16	0.44
TB mortality rate (2015)	0.55	0.36	0.54

R square values derived from linear regression models. In separate models, log-transformed incidence rates and log-transformed TB mortality rates were analyzed. In each model, 2015 rates (either TB incidence or TB mortality) were used to ensure that TB indicators were concordant with other data from which UHC SCI scores were derived.

In sensitivity analyses where TB indicators were removed from the calculation of the UHC SCI, there was no significant differences in the R-squared values compared to those models that included the original UHC SCI estimates. (The R-squared value for TB effective coverage was 0.22 in the model including all countries, 0.15 in high-burden countries and 0.41 in non-high burden countries when the TB variables was excluded from the calculation of UHC SCI; the p-values for these models were all <0.05).

### Dominance analysis

Dominance analysis ranking of relative importance of the 14 indicators differed by stratifications of the UN member states. Across all countries, TB ranked as the eight most dominant variable. In e dominance analysis stratified by TB burden, the TB effective treatment coverage estimate was ranked sixth in the 30 high burden countries and ninth in the 153 non-high burden countries (Table 3). However, in separate analyses stratified by world bank income status, TB coverage ranked as third most important variable in LICs and fifth in LMICs and UMICs, but was less important in analysis restricted HICs (ranked seventh).

**Table 3:** Dominance analysis rankings service coverage indicators based as predictors of UHC SCI score, stratified by TB disease burden^a^^,^^b^.

	All countries (n = 183)		High TB burden countries (n = 30)		Non-High TB burden countries (n = 153)	
	Std. Dominance Coefficient	Rank	Std. Dominance Coefficient	Rank	Std. Dominance Coefficient	Rank
Family planning	0.09	4	0.10	3	0.09	4
Pregnancy and delivery care	0.1	3	0.07	7	0.10	3
Child immunization	0.06	11	0.08	6	0.06	11
Child pneumonia treatment	0.08	7	0.05	10	0.08	7
HIV treatment	0.06	10	0.04	11	0.06	10
Tuberculosis effective treatment	0.06	9	0.07	8	0.06	9
Water and sanitation	0.13	2	0.15	1	0.13	2
Prevention of cardiovascular disease	0.08	8	0.05	9	0.09	6
Management of diabetes	0.01	13	0.00	13	0.01	13
Tobacco Control	0.01	12	0.02	12	0.01	12
Hospital access	0.09	5	0.09	5	0.09	5
Health care worker density	0.14	1	0.13	2	0.14	1
Health Security	0.08	6	0.1	4	0.08	6

^a^Malaria prevention is also included in the UHC SCI for countries where malaria is prevalent. Since most countries do not collect this data, we excluded it from our analysis.

^b^ Cervical cancer screening and access to essential medicines were excluded because of low data availability

## Discussion

In this study we sought to investigate whether indicators of TB epidemiologic control can serve as useful surrogates for more complex indices of UHC, such as the UHC SCI, especially in countries with a high TB burden. As evidence of the utility of TB indices as tracers of broader health service coverage, both TB incidence and TB mortality rates were strongly and inversely correlated with UHC SCI scores. Furthermore, in regression analysis including all 183 countries, TB incidence was associated with 45% of the variability in the UHC index and TB mortality rates accounted for 55% of UHC SCI variability. Furthermore, in dominance analysis stratified by World Bank income categories, TB ranked among the top five most important indicators in all but the highest income countries. These findings suggest that variability in TB coverage is likely to reflect substantial variability in other dimensions of health service coverage. In countries where TB control efforts are weaker it is probable that other health coverage interventions are less likely to have been instituted.

The SCI index provides a more comprehensive assessment of diverse service coverage components. However, the index is constructed using data of variable quality collected asynchronously. By contrast, the TB indicators assessed in our analysis are collected annually for all countries according to validated methodologies. [8] As such these TB indicators can provide a consistent and reliable assessment of health service coverage, although further prospective analysis is necessary to confirm that country-level trends in TB indices are concordant with trends in UHC SCI. While there is a pressing need for tools for monitoring the coverage of health-care services over time, [12] the complexity of the SCI may limit its reproducibility as an index that can be used to track annual progress across countries. By contrast, TB indicators are collected annually for all countries and, as ecological analyses in Western Europe [13] and Japan [14] have illustrated, rates of change in TB incidence and mortality rates do track with UHC progress at both national and subnational levels over time. Our analysis thus highlights the utility of tracking trends in TB disease burden, in particular TB mortality rates, as a means of tracking progress towards broader UHC targets.

Notably, in analyses stratified by TB burden status, the R-squared values for TB incidence and mortality rates were greater in the non-high TB burden countries than high burden countries; that is, TB indicators explained more of the variability in UHC SCI in non-high TB burden countries than in high TB burden countries. These findings highlight the important relationship between TB burden and UHC, even in non-high burden settings and underscore the importance of ensuring that TB control efforts occur as part of a broader, integrated agenda to improve access to all essential services for all patients. Trends in TB incidence and mortality will only diminish as countries invest in UHC programs that prioritize TB-specific care and prevention efforts,(6, 9) but these programs should be targeted at those populations at highest risk of TB, including people living with HIV, immigrants and those living in congregate settings.[4, 15] To achieve this, the pathway to UHC needs to be a publicly financed approach that covers those core health services that directly benefit people living in poverty [3,16] and TB care and prevention functions must be prioritized within essential service packages.[6, 16]

While TB indicators are important indicators of health coverage equity, the dominance analysis illustrates that several other components of the UHC SCI score are also important drivers of the overall service coverage index. In dominance analysis that included all countries, health care worker density was the highest ranking indicator of UHC SCI, while in analysis restricted to LMIC and UMICs essential medicine provision was highly predictive of the overall SCI score. These findings underscore how achieving UHC in different settings will require different priorities, although an essential package of services is likely to be necessary in all settings.[17] Frameworks that ensure uninterrupted availability of and access to appropriately regulated medications, including anti-tuberculosis medications, as well as the optimal mix of skilled health care workers will be vital in all settings.[18] Tracking these indicators will thus be of high utility as governments and their donor partners look for evidence of progress towards UHC. [13] However, until such indicators are routinely and regularly reported, TB tracer indices, such as those included in our analysis, can serve as a useful ‘canary in the mine’ to highlight when countries are off track towards UHC goals.

Unfortunately, a key challenge with the UHC SCI index is that it is based on a core set of tracer indicators derived from different types and qualities of data on health and health service provision across diverse settings.[5] As noted in the original study [5] describing the genesis and validation of the UHC SCI, data on some of the sub-indices were not available for all countries and a variety of analytical or statistical methods were used to fill gaps, including indicator rescaling, projection to a common year and imputation of missing data. Furthermore, the SCI did not adequately distinguish between countries with the highest level of service coverage provision. In addition, data collection for the individual sub-indices in some countries happened over a timespan of several years, rather than a single point in time. As a consequence, real-time assessments of country-specific scores should be made cautiously. By contrast with the UHC SCI, TB incidence rates and mortality rate are reported every year for every country. Furthermore, these indicators are derived based on validated approaches and using reasonably high quality data. While uncertainties about the quality TB indices exist, especially in settings where national mortality data is estimated from sample subnational vital registration systems, [8] TB indices are reported annually, easily accessible and subject to rigorous interrogation.

This analysis has a number of important limitations. The analysis provides no sub-national granularity and offers no insights into how TB burden disproportionality influences marginalized populations or whether trends in TB indices are concordant with UHC SCI scores at the subnational level. National level TB indicators often offer minimal insight into the kind of sub-national socioeconomic disparities that undermine TB efforts. Furthermore, in many high-income countries that have high UHC SCI scores, TB remains rampant in specific communities, including those dwelling in congregate settings and in immigrant or refugee populations. We recognize that national-level TB indices are crude tools for assessing several coverage disparities in these populations. Nonetheless, in low and middle income countries, and high TB burden countries, trends in TB mortality and incidence may offer useful insights as to overall progress towards achieving universal health coverage. Although allusions are often made to the disproportionate effect of TB on the poorest and socially marginalized groups,[19, 20] robust metrics to quantify risk inequality in TB are lacking. Better tools for tracking TB indicators at subnational level, such as the TB RISK coefficient [21] that can highlight hose TB inequities, may be of value in tracking subnational progress towards UHC in some settings. In addition, the analysis provides no information on trends over time. Prospective analysis using the UHC SCI is limited by the fact that data are not available for all indicators; and reliant on some data that was collected five years ago. Finally, we note that neither UHC SCI nor TB incidence and mortality capture the other dimensions of UHC, namely financial protection and quality of service provision. [22] Metrics that track these will continue to be essential as countries plan towards UHC.

## Conclusion

The UHC SCI has been developed to monitor progress towards the SDG target on UHC, incorporating a diverse set of essential health-care services. As such it serves as a useful point of reference for policy discussions and can help highlight patterns across countries. However, the quality and asynchronous timing of data reporting make tracking country UHC SCI progress over time challenging. By contrast, our analysis illustrates the utility of TB indicators as a means of assessing UHC service coverage. While tracking other indices of health provision is also essential, progress on TB incidence and mortality rates can provide useful equity-oriented health information to inform discussion on whether countries are advancing towards SDG UHC goal.

## References

[1] WHO WB. Tracking universal health coverage: first global monitoring report Geneva: World Health Organization; 2015 [Available from: http://apps.who.int/iris/bitstream/handle/10665/174536/9789241564977_eng.pdf?sequence=1.

[2] Reich MR, Harris J, Ikegami N, Maeda A, Cashin C, Araujo EC, et al Moving towards universal health coverage: lessons from 11 country studies. Lancet. 2016;387(10020):811–6. 10.1016/S0140-6736(15)60002-2 26299185

[3] Stenberg K, Hanssen O, Edejer TT, Bertram M, Brindley C, Meshreky A, et al Financing transformative health systems towards achievement of the health Sustainable Development Goals: a model for projected resource needs in 67 low-income and middle-income countries. Lancet Glob Health. 2017;5(9):e875–e87. 10.1016/S2214-109X(17)30263-2 28728918 PMC5554796

[4] Jamison DT, Summers LH, Alleyne G, Arrow KJ, Berkley S, Binagwaho A, et al Global health 2035: a world converging within a generation. Lancet. 2013;382(9908):1898–955. 10.1016/S0140-6736(13)62105-4 24309475

[5] Hogan DR, Stevens GA, Hosseinpoor AR, Boerma T. Monitoring universal health coverage within the Sustainable Development Goals: development and baseline data for an index of essential health services. Lancet Glob Health. 2018;6(2):e152–e68. 10.1016/S2214-109X(17)30472-2 29248365

[6] Jamison DT, Alwan A, Mock CN, Nugent R, Watkins D, Adeyi O, et al Universal health coverage and intersectoral action for health: key messages from Disease Control Priorities, 3rd edition. Lancet. 2018;391(10125):1108–20. 10.1016/S0140-6736(17)32906-9 29179954 PMC5996988

[7] Watkins DA, Yamey G, Schaferhoff M, Adeyi O, Alleyne G, Alwan A, et al Alma-Ata at 40 years: reflections from the Lancet Commission on Investing in Health. Lancet. 2018;392(10156):1434–60. 10.1016/S0140-6736(18)32389-4 30343859

[8] WHO. Global tuberculosis report 2018 Geneva, Switzerland: WHO; 2018 [updated 2018. Available from: http://www.who.int/tb/publications/global_report/en/.

[9] Participants at the Bellagio Workshop on Implementing Pro-Poor Universal Health C, Bump J, Cashin C, Chalkidou K, Evans D, Gonzalez-Pier E, et al Implementing pro-poor universal health coverage. Lancet Glob Health. 2016;4(1):e14–6. 10.1016/S2214-109X(15)00274-0 26700794

[10] Bank W. World Bank Country and Lending Groups.

[11] Azen R, Budescu DV. The dominance analysis approach for comparing predictors in multiple regression. Psychol Methods. 2003;8(2):129–48. 12924811 10.1037/1082-989x.8.2.129

[12] Bank. WhOatIBfRaDW. Tracking universal health coverage: 2017 global monitoring report. 2017.

[13] Reid MJA, Arinaminpathy N, Bloom A, Bloom BR, Boehme C, Chaisson R, et al Building a tuberculosis-free world: The Lancet Commission on tuberculosis. Lancet. 2019;393(10178):1331–84. 10.1016/S0140-6736(19)30024-8 30904263

[14] Hagiya H, Koyama T, Zamami Y, Minato Y, Tatebe Y, Mikami N, et al Trends in incidence and mortality of tuberculosis in Japan: a population-based study, 1997–2016. Epidemiology and infection. 2018:1–10. 10.1017/S095026881800290X PMC6518835 30409242

[15] Pedrazzoli D, Borghi J, Viney K, Houben R, Lonnroth K. Measuring the economic burden for TB patients in the End TB Strategy and Universal Health Coverage frameworks. Int J Tuberc Lung Dis. 2019;23(1):5–11. 10.5588/ijtld.18.0318 30674374

[16] Gwatkin DR, Ergo A. Universal health coverage: friend or foe of health equity? Lancet. 2011;377(9784):2160–1. 10.1016/S0140-6736(10)62058-2 21084113

[17] Jamison DT, Alwan A, Mock CN, Nugent R, Watkins D, Adeyi O, et al Universal health coverage and intersectoral action for health: key messages from Disease Control Priorities, 3rd edition. Lancet. 2017. 10.1016/S0140-6736(17)32906-9 PMC5996988 29179954

[18] WHO. Implementing the end TB strategy: the essentials. Geneva: WHO; 2015/2016. Report No.: WHO/HTM/TB/2015.31.

[19] Lienhardt C. From exposure to disease: the role of environmental factors in susceptibility to and development of tuberculosis. Epidemiologic reviews. 2001;23(2):288–301. 10.1093/oxfordjournals.epirev.a000807 12192738

[20] Lonnroth K, Castro KG, Chakaya JM, Chauhan LS, Floyd K, Glaziou P, et al Tuberculosis control and elimination 2010–50: cure, care, and social development. Lancet. 2010;375(9728):1814–29. 10.1016/S0140-6736(10)60483-7 20488524

[21] Gomes MGM, Oliveira JF, Bertolde A, Ayabina D, Nguyen TA, Maciel EL, et al Introducing risk inequality metrics in tuberculosis policy development. Nat Commun. 2019;10(1):2480 10.1038/s41467-019-10447-y 31171791 PMC6554307

[22] Creswell J, Jaramillo E, Lonnroth K, Weil D, Raviglione M. Tuberculosis and poverty: what is being done. Int J Tuberc Lung Dis. 2011;15(4):431–2. 10.5588/ijtld.10.0654 21396198

